# Long-term water demand forecasting using artificial intelligence models in the Tuojiang River basin, China

**DOI:** 10.1371/journal.pone.0302558

**Published:** 2024-05-22

**Authors:** Jun Shu, Xinyu Xia, Suyue Han, Zuli He, Ke Pan, Bin Liu

**Affiliations:** 1 College of Management Science, Chengdu University of Technology, Sichuan, China; 2 College of Mathematics and Physics, Chengdu University of Technology, Sichuan, China; UNITEN: Universiti Tenaga Nasional, MALAYSIA

## Abstract

Accurate forecasts of water demand are a crucial factor in the strategic planning and judicious use of finite water resources within a region, underpinning sustainable socio-economic development. This study aims to compare the applicability of various artificial intelligence models for long-term water demand forecasting across different water use sectors. We utilized the Tuojiang River basin in Sichuan Province as our case study, comparing the performance of five artificial intelligence models: Genetic Algorithm optimized Back Propagation Neural Network (GA-BP), Extreme Learning Machine (ELM), Gaussian Process Regression (GPR), Support Vector Regression (SVR), and Random Forest (RF). These models were employed to predict water demand in the agricultural, industrial, domestic, and ecological sectors using actual water demand data and relevant influential factors from 2005 to 2020. Model performance was evaluated based on the Root Mean Square Error (RMSE), Mean Absolute Error (MAE), and Mean Absolute Percentage Error (MAPE), with the most effective model used for 2025 water demand projections for each sector within the study area. Our findings reveal that the GPR model demonstrated superior results in predicting water demand for the agricultural, domestic, and ecological sectors, attaining R^2^ values of 0.9811, 0.9338, and 0.9142 for the respective test sets. Also, the GA-BP model performed optimally in predicting industrial water demand, with an R^2^ of 0.8580. The identified optimal prediction model provides a useful tool for future long-term water demand forecasting, promoting sustainable water resource management.

## Introduction

As a result of the converging forces of global climate change, rapid economic progression, and accelerated urbanization, numerous countries worldwide are grappling with water scarcity issues [1]. In response, the accurate forecasting of future water demand becomes crucial, equipping regional managers to evaluate prospective supply-demand conditions and devise targeted water management approaches to maximize the long-term benefit of water resources [2]. Typically, water demand forecasts are divided into long-term (forecast intervals exceeding two years), medium-term (intervals between three months and two years), and short-term (periods less than three months) [3]. Pertinently, long-term forecasting facilitates the formulation of effective policies and strategies for the operation and management of water supply systems and the identification of beneficial water conservation measures [4]. Factors influencing water demand forecasting are multifaceted, encompassing economic conditions, policy directives, and residential habits. In the context of long-term forecasting, consideration of an expanded set of influences is necessary, potentially inclusive of macroscopic information such as regional economy, climate, and population [5–7]. Historically, while water demand predictions often focused on total regional or seasonal water use [8, 9], they overlooked the significant sectorial variation in water usage.

Agricultural, industrial, domestic, ecological and other sectors consider different influences on water demand, and the exogenous variables of each sector itself (urban population, industrial structure, cropping structure, etc.) are influenced by human planning and activity, which results in water demand forecasting being a complex system with high uncertainty and ambiguity [9, 10]. Methodologies for the study of this complex system can be divided into three categories: traditional qualitative methods, univariate time series models based on statistical methods and multivariate models based on artificial intelligence [11, 12]. While traditional methods such as exponential forecasting [13], quota methods [14] and trend forecasting [15] can accommodate some of the demand forecasting needs, they usually require detailed measurement data and expert knowledge to update model parameters and structure [16]. Traditional statistical models such as the ARIMA model [17] obtain better forecasting results through the relationship between historical and future values of a single factor, which is simple in principle and easy to implement. However, such methods are suitable for short-term forecasting on a weekly, daily or even hourly basis, which requires high smoothness of the time series and cannot handle time series with non-linear characteristics [16]. On the contrary, data-driven artificial intelligence algorithms such as artificial neural networks [18], support vector machines [19], system dynamics [20] and Kalman filtering [21] are able to explore the logical relationships within the data, with the advantages of high prediction accuracy and fast computing speed. BP neural networks are one of the relatively mature network structures in ANNs. Wu [22] conducted an empirical study on water demand forecasting in Taiyuan City and found that the PCA-BP model was superior in forecasting accuracy to models such as ARIMA, Grey-Markov, and serial regression. Despite its relatively recent introduction, ELM has also been introduced into various fields of forecasting applications. Deo and Şahin [23] showed that the ELM model developed for predicting the monthly effective drought index significantly outperformed the ANN model. Adnan [24] et al. have successfully applied ELM combined with SAMOA, PSOGWO and other meta-heuristic optimization algorithms for monthly flow prediction from local hydrometeorological data. However, the employment of ELM models in water demand forecasting is less common [25]. Methods demanding larger sample sizes often struggle to handle the intricate non-linear mapping relationships between water demand and its influencing factors under conditions of limited samples, given the inherent non-linear and non-smooth characteristics of the available data in the water demand forecasting process [26]. SVR methods, grounded in the principle of structural risk minimization rather than empirical risk minimization [27], resist over-learning when trained on small samples, thereby demonstrating robust generalization capabilities. It has been substantiated that establishing appropriate model parameter settings can significantly enhance SVR prediction accuracy [28]. Despite this, both the ANN and SVR models above function as black box models, meaning they can predict target variable values based on the data, but they cannot illuminate the underlying rules or patterns within the model [29]. In contrast, decision tree algorithms, including Random Forest (RF), offer an alternative to black box models by providing a systematic graphical representation of explanatory variables and their critical values. This approach can effectively distinguish sub-populations with differing behaviors in the target variable (in this case, water demand). RF, in particular, represents an exemplary manifestation of such algorithms and has proven effective in predicting water demand in high-dimensional data [30]. Furthermore, researchers have recently shown increasing interest in probabilistic forecasting methods [31, 32]. Gaussian Process Regression (GPR), a probabilistic forecasting algorithm emerging from statistical learning and Bayesian theory, possesses robust generalization capabilities for modelling interval predictions, handling missing and anomalous data, as well as dealing with both high and small sample issues [33].

Despite the validation of each of the aforementioned methodological models as reliable for water demand forecasting, it is important to note that no single model is universally superior to all others in all cases. It remains necessary to examine each region independently to assess the merits of each model or combination of methods [26]. Therefore, this study aims to compare the applicability of five selected models (GA-BP, ELM, GPR, SVR and RF) for predicting water demand in different water use sectors. The aim is to identify the most appropriate forecasting models for each water use type, taking into account its inherent characteristics and the non-linear relationship between influencing factors and water demand. This will facilitate reliable water demand forecasting for 2025 and provide a basis for regional water resource management. The Tuojiang River Basin in Sichuan Province serves as the study area for this paper, with key influencing factors extracted for four water use sectors: agriculture, industry, domestic and ecological. We aim to build a water demand forecasting model based on the above five AI techniques. The optimal model for each water use sector will be selected using RMSE, MAPE and R^2^ as evaluation indicators. These will be used to complete the water demand forecasts for the study area in 2025. The paper is organised as follows: Section 1 provides an overview of the current state of research in water demand forecasting. Section 2 provides an overview of the study area. Section 3 establishes water demand impact indicators and briefly describes the underlying principles of the methodology used in this study. Section 4 is the results. Section 5 provides a series of discussions. Section 6 presents the conclusions.

### Study area

**Fig 1:**
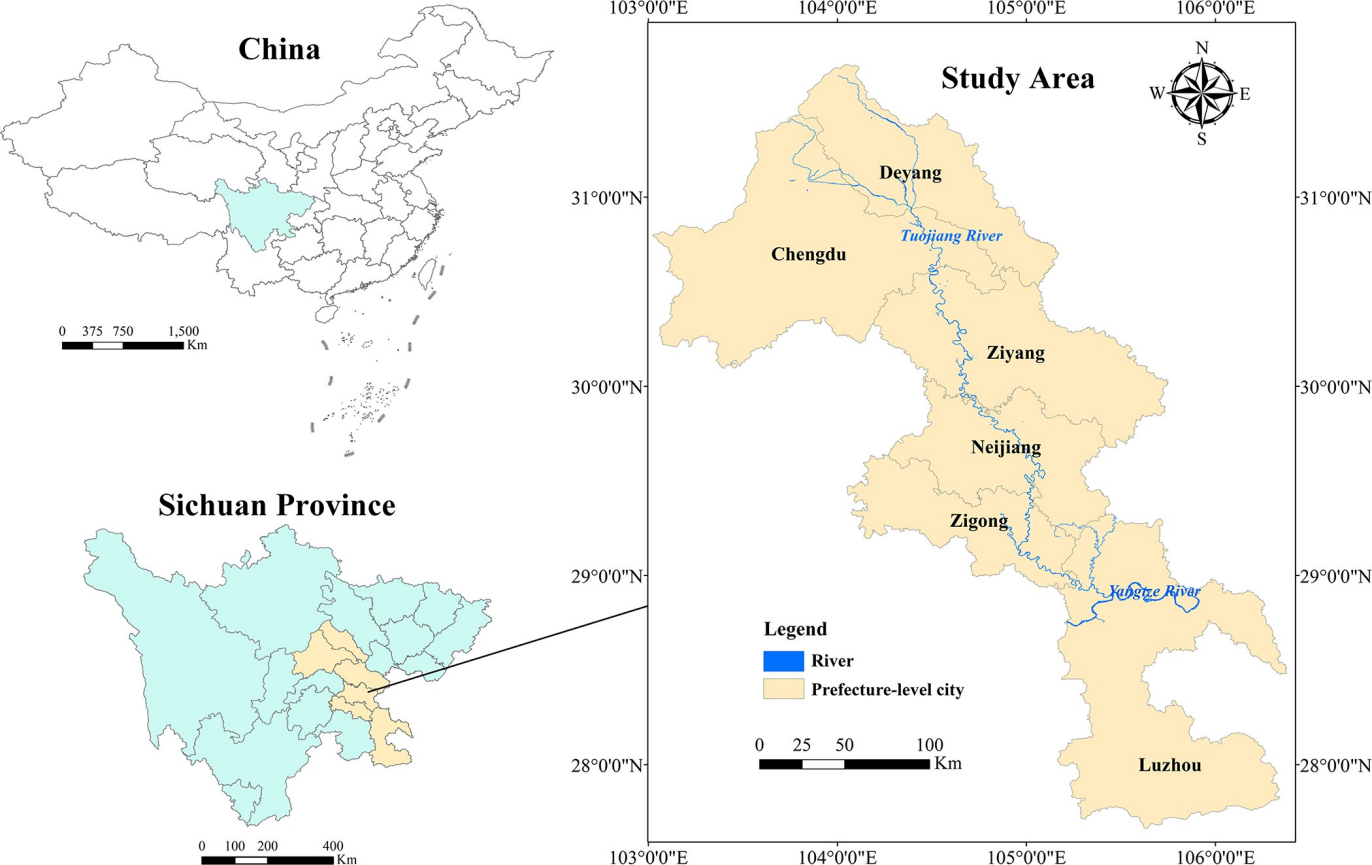
Map of Tuojiang River basin (the map was created by our team using standard maps obtained from the Ministry of Natural Resources of the People’s Republic of China (https://www.mnr.gov.cn/) using ArcMap 10.8). These maps with approval numbers can be accessed free of charge from the agency’s public website GS(2023)2765 (http://bzdt.ch.mnr.gov.cn /browse.html?picId = %224o28b0625501ad13015501ad2bfc2190%22), Sichuan S(2021)00056 (https://scsm.mnr.gov.cn/StandMaps/mapDetails.html).

The Tuojiang River basin ($103^{{}^{\circ}}41\prime E∼105^{{}^{\circ}}55^{\prime }E$, $28^{{}^{\circ}}50^{\prime }N∼31^{{}^{\circ}}41^{\prime }N$) (Fig 1), situated in southwest China, is a first-order tributary of the Yangtze River spanning a length of 627.4 km and covering an area of 27 km^2^. The main regions through which the Tuojiang River flows include Deyang, Chengdu, Ziyang, Neijiang, Zigong and Luzhou. It provides an important source of water. The terrain of the basin varies from high in the northwest to low in the southeast, displaying a landscape characterized by northern mountainous areas, central plains and low hills, and southern hills. The region’s favorable topographic and climatic conditions support a robust industry and high levels of socio-economic activity, resulting in significant water demand. Simultaneously, the relatively complex natural environment fosters variability in the socio-economic development and industrial structures of the different regions, rendering the Tuojiang River basin an ideal study area for water demand forecasting.

### Data and methodology

**Fig 2:**
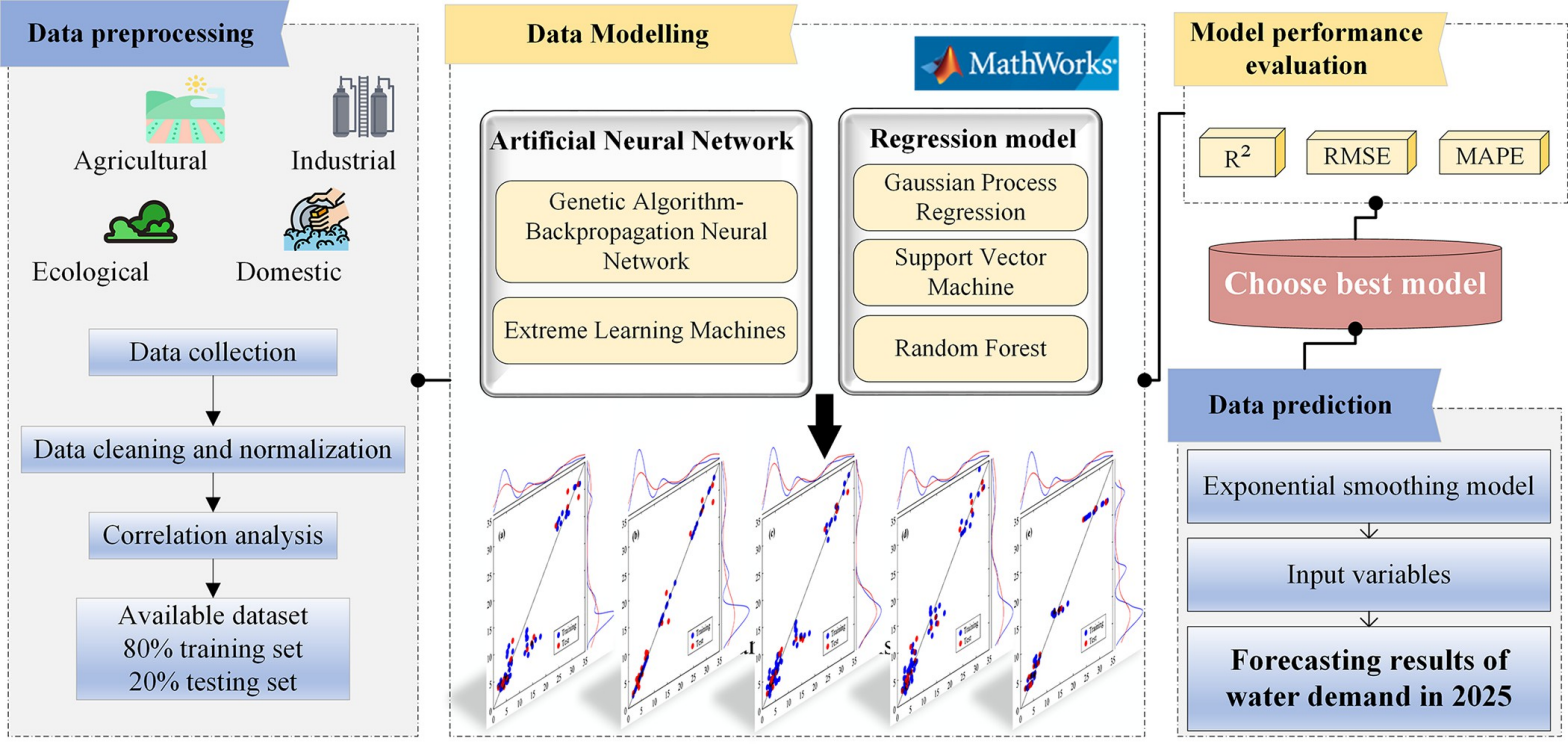
Overall methodological framework.

This research compared the applicability of two types of methods for water demand forecasting in different water use sectors. They are artificial neural network models (GA-BP, ELM) and nonlinear regression models (GPR, SVR, RF). Fig 2 illustrates the methodology applied in this study. Firstly, data preparation for the study area included establishing the indicator system and collecting data corresponding to the input and output variables. This was followed by data cleaning, regularization, and division into an 80% training set and a 20% testing set. Secondly, Matlab software was utilized to conduct modeling and simulation analyses of the five method categories for different water use sectors. This enabled the acquisition of predicted values for the training and testing sets, and visualization of the fit. The performance of the model methods was then evaluated using R^2^, RMSE, and MAPE as metrics, leading to the selection of an optimal model for each water use sector. Finally, the smoothing index model, a traditional time series forecasting model, was employed to predict the 2025 impact factor data as input variables for the optimal model. This allowed the prediction of the 2025 agricultural, industrial, domestic, and ecological water demands for the study area.

### Data collection and indicators extraction

**Fig 3:**
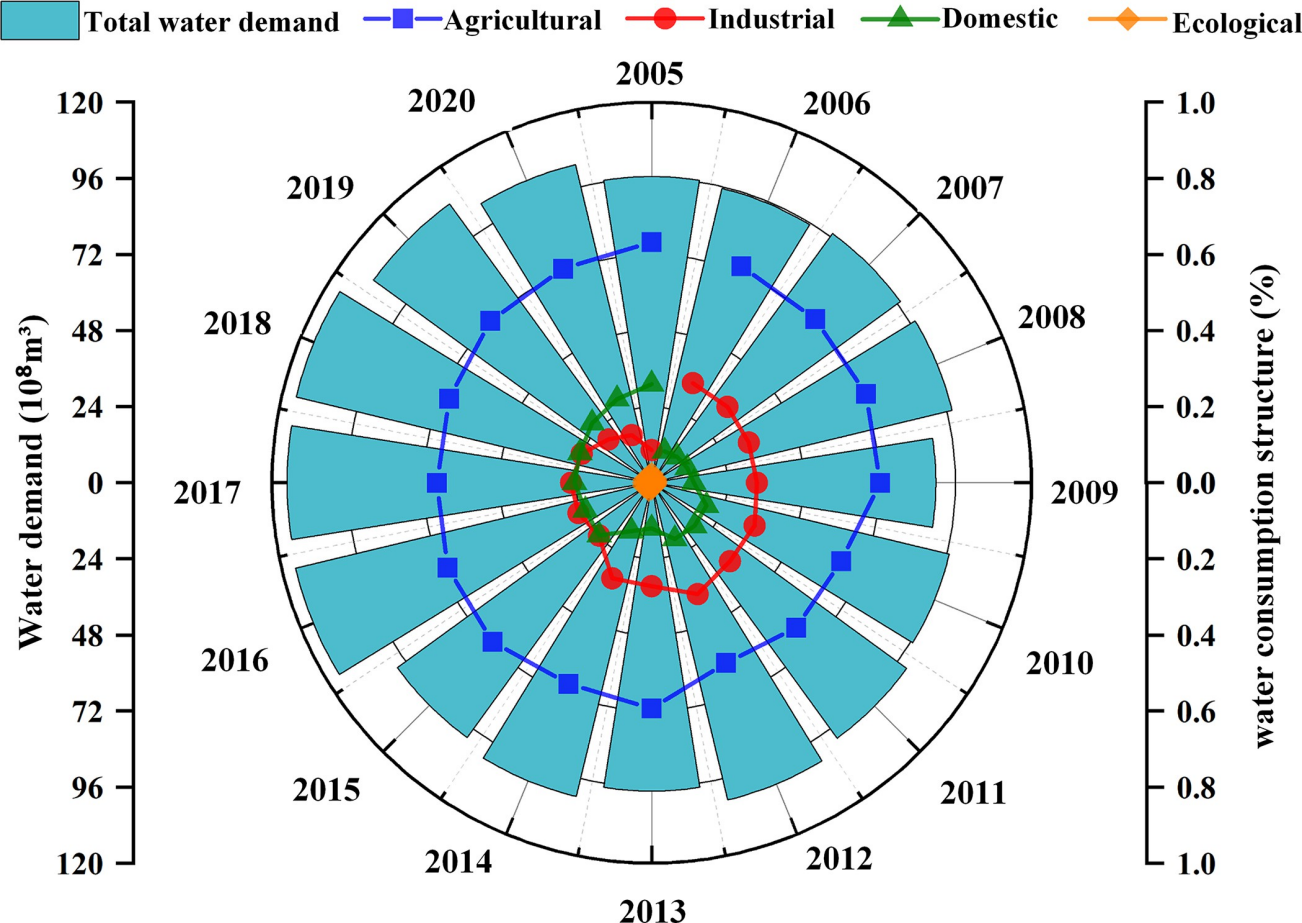
Water demand in the Tuojiang River basin from 2005 to 2020.

The data employed in this research comprises annual water demand for agricultural, industrial, domestic, and ecological sectors across six regions in the Tuojiang River Basin, spanning the period 2005 to 2020. The full set of data collected can be found in the first worksheet of the S1 Table. Fig 3 illustrates the variability in water demand within this timeframe for the entire basin, accompanied by data on the structure of water consumption. The Tuojiang River Basin’s total water demand exhibits minor fluctuations between 2005 and 2015, roughly 96×10⁸m^3^, punctuated by a surge in demand from 2016 to 2018, followed by a declining trend in 2019 and 2020. In terms of water consumption composition, agriculture claims the most substantial and relatively steady share, approximately 60%, succeeded by industrial, domestic, and ecological uses. Industrial water use displays a year-on-year decrease, while domestic water use manifests a pronounced ascending trend. The overall proportion of ecological water usage remains within a narrow range over the years.

Secondary indicators for forecasting water demand in the four sectors—agricultural, industrial, domestic, and ecological—were derived from previous research findings as presented in Table 1. Descriptive statistics of all indicators are shown in Table 2. Upon data collection, further relevant analysis was carried out, as detailed in the RESULTS section.

**Table 1:** Water demand forecasting indicator system.

Variables	Definition	Positive or negative correlation	References
Agricultural water demand (*AWD*)	Agricultural water includes irrigation of cultivated land and forest land, garden land, pasture irrigation, fish pond replenishment and livestock water.		
Agricultural EVA (*A*_1_)	Reflects the final results of agricultural production and business activities and their contribution to society.	+	[34]
Area of irrigated arable land (*A*_2_)	An important indicator that shows the construction of regional farmland water resources.	+	
Area of total crop sown (*A*_3_)	The entire area of land it is evaluated, including arable or non-arable, where crops have been sown or transplanted for harvest within the calendar year.	+	
Percentage of primary industries (*A*_4_)	Indicates the industrial structure of the regional primary industries, which are predominantly agricultural industries.	-	[35]
Industrial water demand (*IWD*)	Industrial water refers to the water used by industrial and mining enterprises in the production process for manufacturing, cooling and other aspects of water, excluding the reuse of water inside the enterprise		
Percentage of secondary industries (*I*_1_)	Represents the industrial structure of the regional secondary industries dominated by industrial.	-	[20]
Industrial EVA (*I*_2_)	It is the aggregate indicator on which the rate of industrial development is calculated.	+	[36]
Operating income of industrial enterprises above designated size (*I*_3_)	This indicator represents the industrial legal entities with RMB 20 million or more income from the primary business or the total business income. The water demands for production processes of these industries dominate the regional industrial water demand.	+	[37]
Number of industrial households above designated size (*I*_4_)		+	
Domestic water demand (*DWD*)	Domestic water use includes urban domestic water use and rural domestic water use, of which urban domestic water use consists of residential water use and public water use.		
Per capita GDP (*D*_1_)	It is an important indicator of the level of economic development and affluence of a country or region.	+	[38]
Population density (*D*_2_)	Population per unit area at the end of the year represents the population distribution.	+	
Urbanization rate (*D*_3_)	Demographic indicators are used to determine the degree of urbanization.	-	[39]
Urban per capita disposable income (*D*_4_)	The sum of the final consumption expenditures and savings of urban residents reflects the living standards of urban and rural residents.	+	
Rural per capita disposable income (*D*_5_)		+	
Ecological water demand (*EWD*)	Ecological water only includes water supplied for the urban environment and part of the recharge of rivers, lakes and wetlands, and does not include the amount of water naturally met by precipitation and runoff.		
Runoff depth (*E*_1_)	Represents the annual runoff depth derived from the natural runoff of rivers, lakes, glaciers, and other surface water bodies.	+	[40]
Urban green space (*E*_2_)	The general term for vegetated land, open land, and water body in an urban planning area indicates the urban environment’s water demand. It determines the human measures for water supply.	+	[41]
Area of road sweeping and cleaning (*E*_3_)	Indicates the level of development of urban environmental governance, which increases with increase of the size of the city.	+	
Rainfall (*E*_4_)	The water requirement of the ecological environment can be influenced to some degree by meteorological data, such as temperature and precipitation. Rainfall amount affects the amount of water supply to the ecological environment, whereas temperature indirectly affects the ecological water requirement by modulating factors like evaporation and the ecosystem’s living style.	+	[38]
Average temperature (*E*_5_)		+	

**Table 2:** Descriptive statistics of data.

Variables	Unit	Minimum	Maximum	Mean	Standard Deviation
*AWD*	10^8^m^3^	1.89	32.91	9.92	9.07
*A*_1_	Billion yuan	46.73	655.17	184.81	123.05
*A*_2_	10^3^hm^2^	74.09	372.11	163.25	82.47
*A*_3_	10^3^hm^2^	283.10	835.13	521.87	148.67
*A*_4_	%	0.03	0.35	0.15	0.06
*IWD*	10^8^m^3^	0.21	15.86	3.97	4.08
*I*_1_	%	0.28	0.62	0.50	0.08
*I*_2_	Billion yuan	37.11	5418.50	919.89	1279.18
*I*_3_	Billion yuan	169.68	14966.49	2476.88	3232.56
*I*_4_	PCS	11.00	4308.00	1082.03	1093.78
*DWD*	10^8^m^3^	0.74	16.59	2.84	3.63
*D*_1_	People/km^2^	6014.00	85679.00	32150.50	18966.60
*D*_2_	yuan	345.90	1461.00	653.47	249.68
*D*_3_	%	0.33	39.34	3.80	9.88
*D*_4_	yuan	6906.31	48592.50	22821.03	10436.82
*D*_5_	yuan	2986.80	26431.74	10091.59	5619.95
*EWD*	10^8^m^3^	0.01	1.69	0.27	0.40
*E*_1_	mm	140.80	1013.50	455.71	191.87
*E*_2_	10^3^hm^2^	398.00	42768.33	6585.68	9312.94
*E*_3_	10^3^hm^2^	121.00	16684.58	1867.76	3372.75
*E*_4_	mm	520.30	1610.70	991.46	208.00
*E*_5_	°C	15.90	19.30	17.60	0.83

### Predictive models

This section briefly describes the principle and theoretical background of GA-BP due to space limitation, the rest of the methods (ELM, GPR, SVR, RF) are well known and are provided only for the reference of interested readers: ELM [42], GPR [33], SVR [43], RF [44].

**Fig 4:**
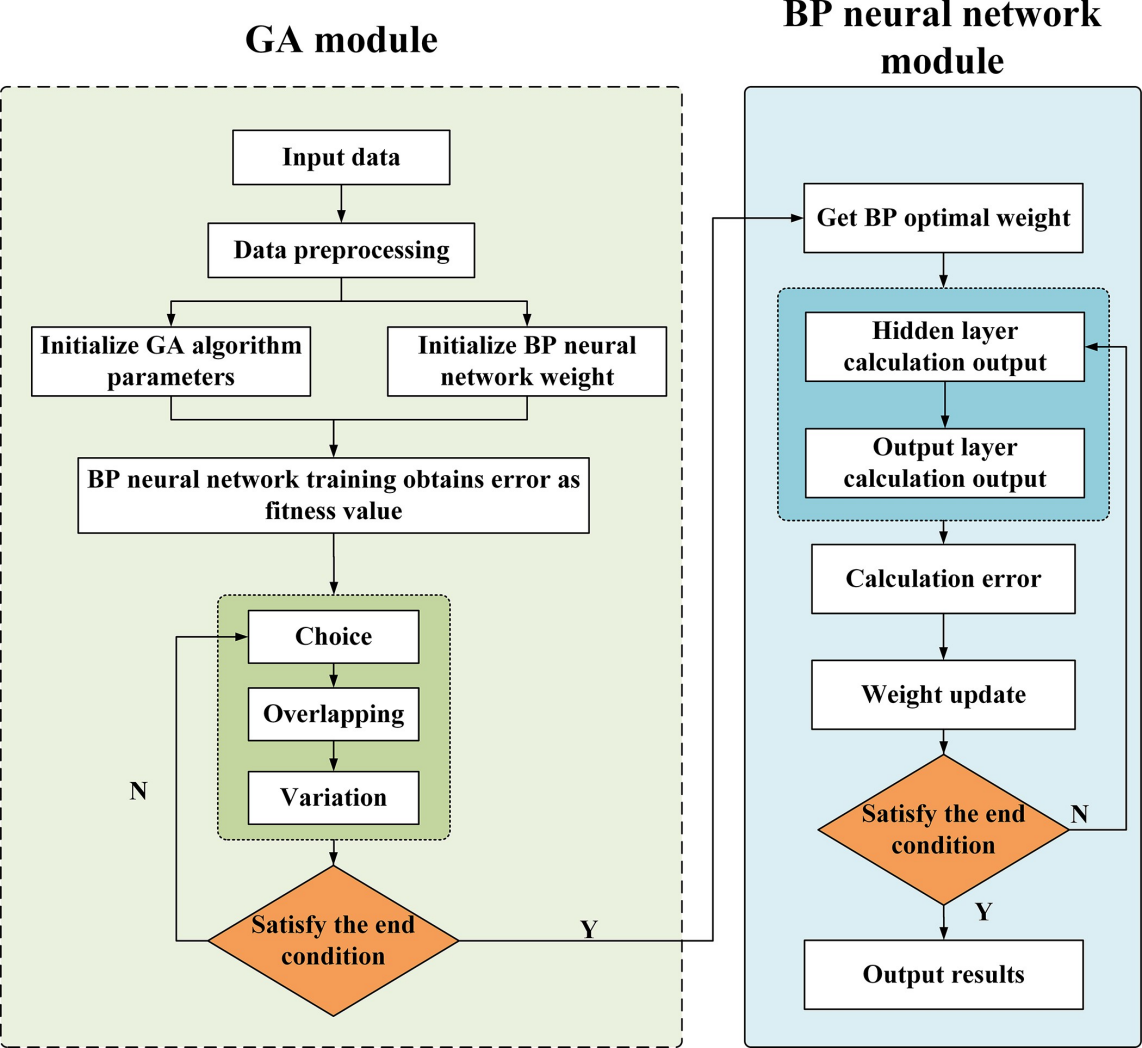
Flow chart of the GA-BP neural network.

The BP Neural Network is a class of neural networks wherein the input sample data is processed forward and the output error is propagated backward. The BP neural networks acquire a priori knowledge via a search procedure targeting the ideal weight set for neuronal connections and threshold values [45]. The recurrent updating of connection weights may be overseen by GA, a global optimization algorithm grounded in evolutionary and natural selection principles, eventually leading to the creation of a set of BP weights conducive to optimal or near-optimal performance of the network structure [46]. The algorithmic flowchart of the GA-BP is shown in Fig 4 below.

### Model performance and evaluation

To evaluate the accuracy of the prediction model, statistical indices such as R^2^, RMSE, and MAPE are employed [13], with their respective calculation formulae provided below:

$$RMSE=\sqrt{\frac{\sum_{k=1}^{n}{\left(y_{k}-{\hat{y}}_{k}\right)}^{2}}{n}}$$ (1)

$$MAPE=\frac{1}{n}\sum_{k=1}^{n}\left|\frac{y_{k}-{\hat{y}}_{k}}{y_{k}}\right|\times 100\%$$ (2)

$$R^{2}=1-\frac{\sum_{k=1}^{n}{\left(y_{k}-{\hat{y}}_{k}\right)}^{2}}{\sum_{k=1}^{n}{\left(y_{k}-\bar{y}\right)}^{2}}=1-\frac{RMSE}{Var}$$ (3)

Where, *y*_*k*_ represents the true value of the dependent variable, $\bar{y}$ represents the sample mean, ${\hat{y}}_{k}$ represents the predicted value, and *n* represents the number of samples. MAPE employs percentages to gauge the magnitude of deviations, offering ease of understanding and interpretation, and is less susceptible to extreme values. RMSE measures the discrepancy between predicted and true values. It is sensitive to outliers in the data and needs to be paired with the magnitude of true values for interpretation. R^2^ indicates the goodness of fit; the closer the value is to 1, the higher the explanation power of the independent variable on the dependent variable.

## Results

This section presents the results of five distinct models—GA-BP, ELM, GPR, SVR, and RF—employed for forecasting water demand across four different water use sectors. Detailed numerical results can be found in the second through sixth worksheets of the S1 Table. Each model’s performance was assessed using statistical parameters. In each method, 80% of the historical annual water demand data was allocated to the training set, with the remaining 20% assigned to the test set. The gathered data was segmented into four categories: agricultural, industrial, domestic, and ecological. Each category consisted of 16 years of water demand data from six regions, yielding a total of 96 data points. Out of these, 75 data points were used for the training phase, and 21 were used for testing. Prior to model construction, Pearson correlations between the input and output sets were taken into account.

### Data preprocessing

**Fig 5:**
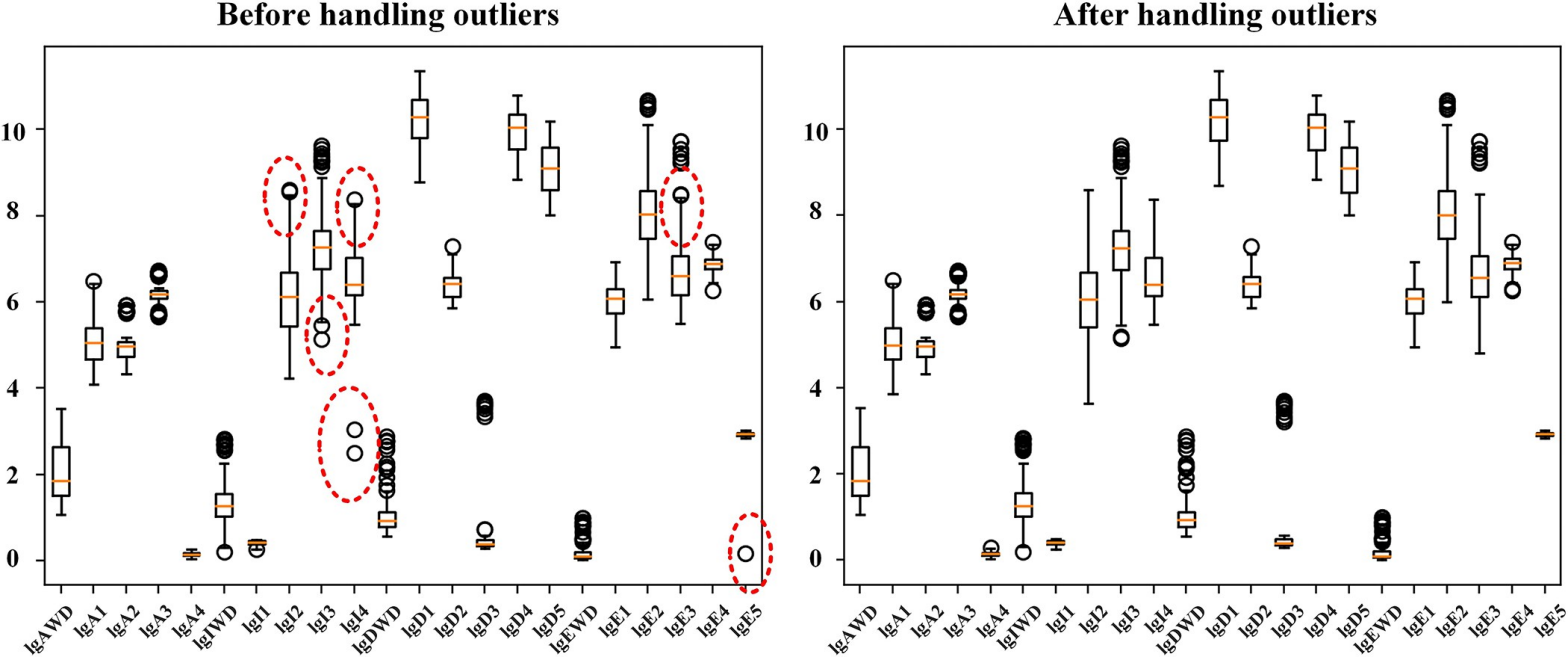
Comparison of boxplot before and after outlier processing.

Before bringing normalized data into the model for training and testing, it is necessary to clean the data to remove outliers. Outliers may lead to overfitting of the model, but there is a high degree of variability in the level of socio-economic development of the six cities within the Tuojiang River Basin, making it highly probable that there are extreme values in the dataset that contain important information. In order to avoid removing normal extreme values, this study used multiple outlier detection methods, integrating three outlier detection methods, Isolation Forest, Elliptic Envelope, and One-Class SVM, using the vote method to determine the final outliers and replacing the outliers with the mean value. The boxplot provided an overall overview of the data distribution before and after outlier processing, as shown in Fig 5.

### Factors affecting water demand

**Fig 6:**
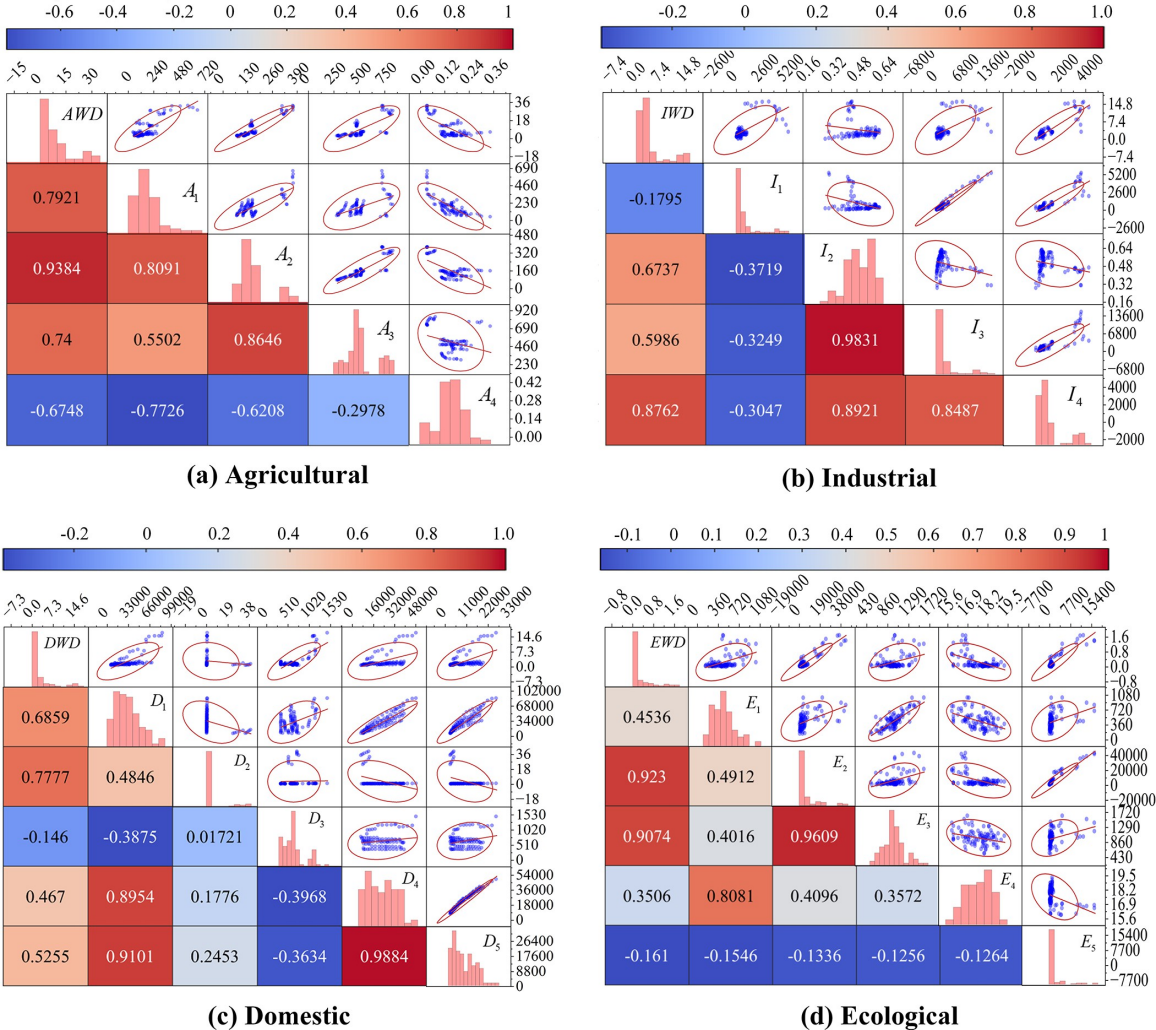
Correlation heatmap of parameters influencing water demand.

Correlation analysis was performed on the data to understand the relationship between the different input variables and the dependent variable. Pearson correlation analysis was performed on agricultural, industrial, domestic, and ecological water demand, and their selected 4–5 influencing factors. The scatter matrix plots of all the characterized data were plotted to further understand the distribution of the data for each indicator and their relationship with the water demand, and the associated heat map obtained is shown in Fig 6.

The results indicate that in agricultural terms, the area of irrigated arable land (*A*_2_) has the most significant impact (0.9384) and is positively correlated with water demand. This result, along with agricultural EVA (*A*_1_)and the area of total crop sown (*A*_3_), each with Pearson coefficients above 0.7, confirms that these variables are the primary factors influencing agricultural water demand. This finding is consistent with the reality that irrigation accounts for a substantial proportion of water usage in agriculture. Industrial water use is positively correlated with the number of industrial enterprises above a designated size (*I*_4_) (0.8762), industrial EVA (*I*_2_) (0.6737), and the operating income of industrial enterprises above designated size (*I*_3_) (0.5986), in descending order of correlation. Given that large-scale industrial enterprises typically require substantial water resources for their production processes, it is reasonable to utilize these relevant statistical indicators as input variables for predicting industrial water demand. In addition, the negative correlation observed between the percentages of primary and secondary industries (*A*_4_, *I*_1_) on local agricultural and industrial water demand can be attributed to the more noticeable regional differences in agricultural and industrial water demands. Their industrial share statistics are based on the local industrial structure, and sample points with higher water demand and smaller industrial share data lead to a negative Pearson correlation coefficient. However, the absolute values of these correlation coefficients suggest that both input parameters maintain a degree of correlation with their dependent variables and remain beneficial when used as input parameters for artificial intelligence models. In terms of domestic water use, population density (*D*_2_) (0. 7777), a statistical indicator indicating the spatial distribution of the population, shows a significant positive correlation with the dependent variable. Furthermore, indicators linked to quality of life, such as per capita GDP (*D*_1_) (0.6859), urban per capita disposable income (*D*_4_) (0.467) and rural per capita disposable income (*D*_5_) (0.5255), demonstrate a substantial positive correlation. Conversely, the urbanization rate (*D*_3_) (-0.146), a measure of urbanization level, is negatively correlated with domestic water use. Ecologically, the correlation between the area of urban green space (*E*_2_) (0.9230) and the area of road sweeping and cleaning (*E*_3_) (0.9074), both requiring human intervention to supply water, holds high significance in relation to ecological water demand. In contrast, runoff depth (*E*_1_), rainfall (*E*_4_) and average temperature (*E*_5_), as several meteorological factors with greater uncertainty, indirectly influence ecological water demand by affecting the amount of ecological water available, evapotranspiration, and the living conditions of the ecological environment. This is reflected in the relatively weak correlation between these three input variables and the dependent variable. In summary, most of the input variables extracted for this study show a significant correlation with the output variables. Although some factors exhibit a relatively slight effect on the target variables, all parameters were used as inputs due to the low dimensionality of the extracted indicator characteristics, which do not increase the computational complexity of the model.

### Comparison of model performance

In this study, Matlab2020a was used to construct the model. In the training stage, Mean Squared Error is adopted as the fitness of algorithm update, and the main parameters of the algorithm were set as shown in Table 3.

**Table 3:** Main parameter of the artificial intelligence models.

Algorithm	Parameter	Detailed description			
GA	Name	Maxgen	Pop	Ub	Lb
	Value	10	50	[20,20]	[1,1]
BP	Name	LearningRate	LearningGoal	HiddenNode	Epoch
	Value	0.01	0.01	9	5000
ELM	Name	NumHiddenLayer	HiddenNode		
	Value	1	12		
SVR	Name	KernelFunction			
	Value	polynomial			
GPR	Name	KernelFunction	BasisFunction	Standardize	
	Value	squaredexponential	Constant	True	
RF	Name	Method	NumLearningCycles	MinleafSize	
	Value	Bag	10	8	

### Agricultural factors

**Fig 7:**
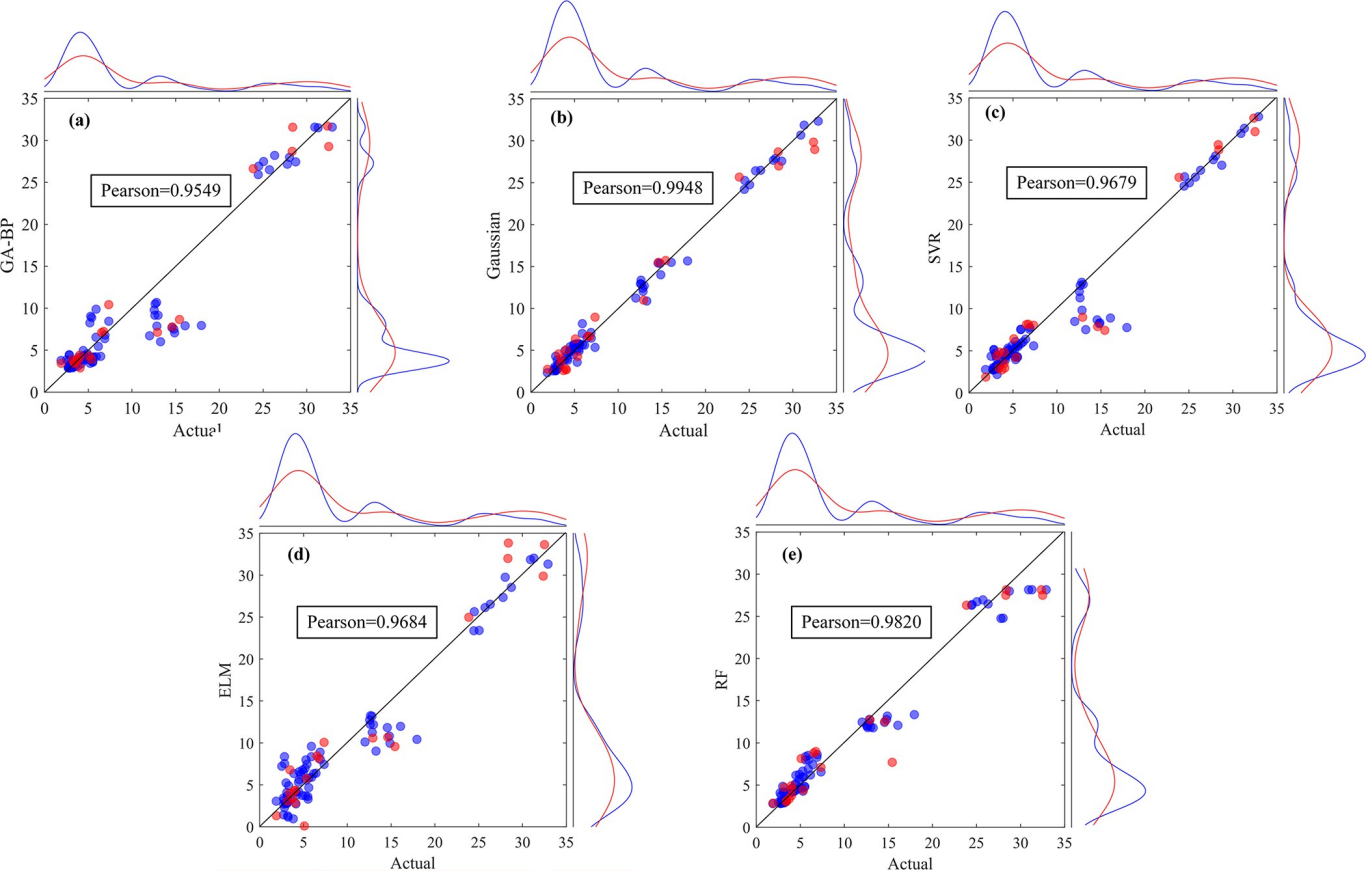
Comparison of predicted and actual values based on (a)GA-BP; (b)GPR; (c)SVR; (d)ELM; (e)RF for agricultural water demand.

The fitting results for agricultural water demand prediction from each approach are shown in Fig 7. The optimal model is further selected for quantitative evaluation using the established evaluation indicators (Table 4).

**Table 4:** Performance evaluation results for each model for agricultural water demand.

		GA-BP	GPR	SVR	ELM	RF
**Training set**	R^2^	0.8921	**0.9924**	0.9313	0.9385	0.9675
	RMSE	2.8168	**0.7456**	2.2473	2.1264	1.5454
	MAPE	22.5818	**8.2491**	15.7227	28.3645	14.5446
**Test set**	R^2^	0.9247	**0.9811**	0.9359	0.9304	0.9392
	RMSE	2.8628	**1.4338**	2.6419	2.7525	2.5724
	MAPE	19.9185	**16.7736**	20.4280	25.1140	20.2835

Fig 7 illustrates the deviation between the predicted and actual water demand in both the training and test sets. A closer proximity of data points to the diagonal line signifies a better fit from the model. Each of the five models yields a Pearson correlation coefficient greater than 0.9, signifying a robust correlation between the predicted and actual values. Of these, the GPR model exhibits a stronger concentration of data points on the diagonal for both the training and test sets, and its Pearson correlation coefficient of 0.9948 is the highest among the five models.

Upon comparisons of R^2^, RMSE, and MAPE values among the five models, the bold data in the Table signify the optimal model under each evaluation indicators. It’s evident that the three evaluation indicators indicate the best performance of the GPR model in both the training and testing stages. When combined with insights obtained from Fig 7, it’s evident that the GPR model outperforms the other four models in predicting agricultural water demand.

### Industrial factors

**Fig 8:**
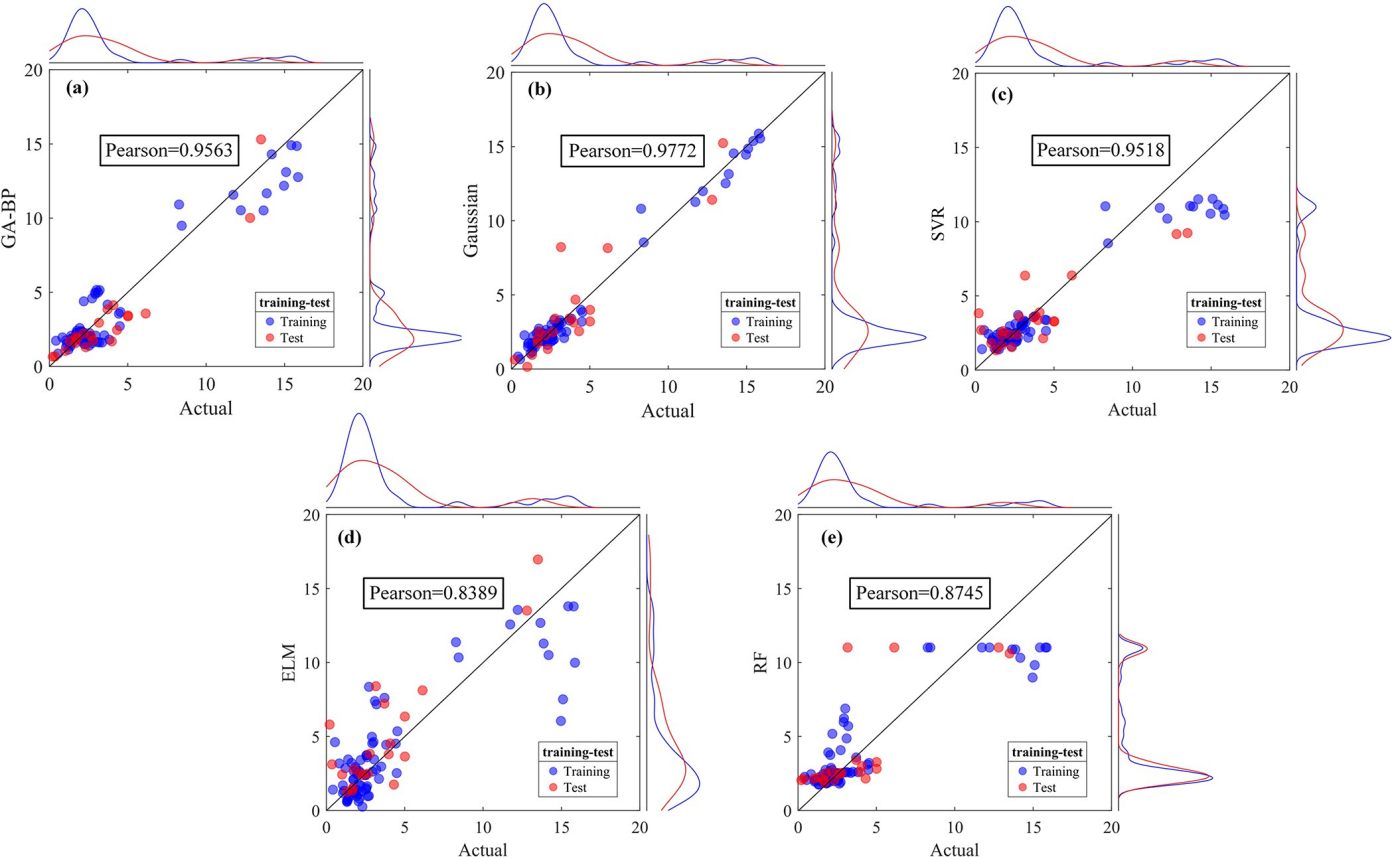
Comparison of predicted and actual values based on (a)GA-BP; (b)GPR; (c)SVR; (d)ELM; (e)RF for industrial water demand.

The fitting results of various models for the prediction of industrial water demand are illustrated in Fig 8. The optimal model is selected by further quantitative evaluation using the evaluation indicators (Table 5).

**Table 5:** Performance evaluation results for each model for industrial water demand.

		GA-BP	GPR	SVR	ELM	RF
**Training set**	R^2^	0.9192	**0.9809**	0.8773	0.7207	0.8006
	RMSE	1.2080	**0.5878**	1.4892	2.2467	1.8982
	MAPE	32.6387	**19.2381**	30.4944	59.3711	46.2197
**Test set**	R^2^	**0.8580**	0.8074	0.6929	0.5486	0.4973
	RMSE	**1.2828**	1.4939	1.8865	2.2874	2.4137
	MAPE	**34.4476**	48.7263	139.8812	198.7939	106.5502

The principle of Fig 8 is the same as that of section 4.2. Despite the GPR model having the highest Pearson coefficient at 0.9772, several data points from the testing set substantially deviate from the diagonal. The data point distribution in the GA-BP and ELM models is similar, with Pearson coefficients around 0.96, but the GA-BP model shows a higher concentration of data points for smaller values.

From Table 5 it can be seen that the GPR model performs best in the training stage, with an R^2^ of 0.9809. However, in the testing stage, the R^2^ for the GPR model drops to 0.8074, which is lower than that of the GA-BP model. Considering the performance of each model in both training and testing, the GA-BP model exhibits greater stability. In conjunction with the model performance displayed in Fig 8, the GA-BP model is determined to be the best for predicting industrial water demand.

### Domestic factors

**Fig 9:**
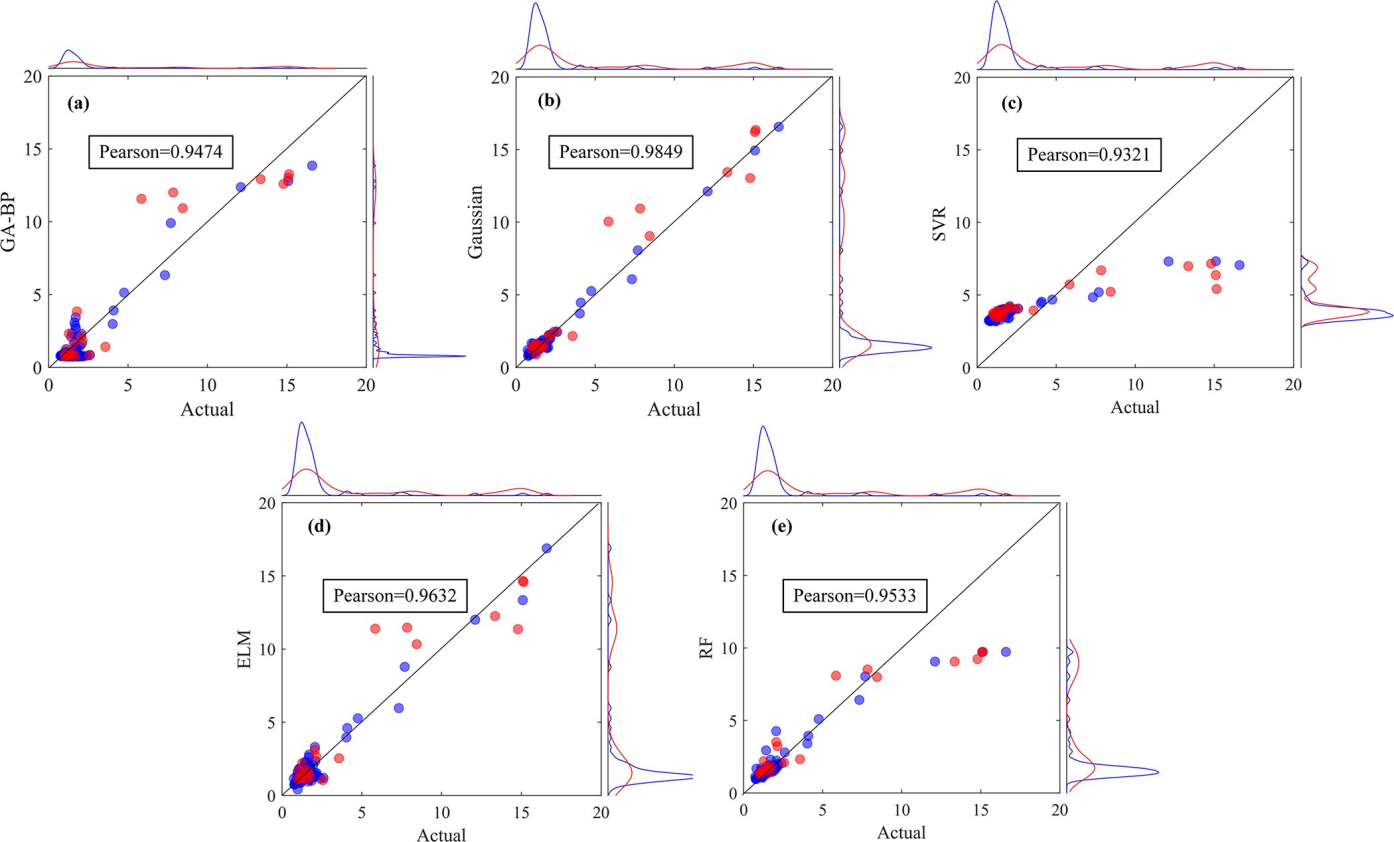
Comparison of predicted and actual values based on (a)GA-BP; (b)GPR; (c)SVR; (d)ELM; (e)RF for domestic water demand.

The fitting results of various models for domestic water demand prediction are shown in Fig 9. The optimal model is chosen for further quantitative evaluation using the designated evaluation indicators (Table 6).

**Table 6:** Performance evaluation results for each model for domestic water demand.

		GA-BP	GPR	SVR	ELM	RF
**Training set**	R^2^	0.9050	**0.9902**	0.1124	0.9567	0.8335
	RMSE	0.8654	**0.2779**	2.6456	0.5843	1.1458
	MAPE	33.8193	**11.8243**	157.5971	25.8852	19.2321
**Test set**	R^2^	0.8470	**0.9338**	0.3651	0.8769	0.7823
	RMSE	2.0001	**1.3152**	4.0745	1.7938	2.3860
	MAPE	37.4985	**19.9105**	113.1570	29.1469	28.9797

Fig 9 shows the same principle as discussed in section 4.2. From the distribution of data points in relation to the position of the diagonal line, it can be seen that for data points with smaller actual values, the models, except for the SVR model, fit the actual values better in both the training and testing phases. However, for the data points with larger values located in the upper right part of the distribution, the data points deviated from the diagonal to a greater extent in the rest of the models except for the GPR model, especially in the test set. Meanwhile, the GPR model has the largest Pearson coefficient value.

As seen in Table 6, the three evaluation indices indicate the optimal performance of the GPR model, both in the training and testing phases. Coupling this with insights from Fig 9, it’s discernible that the GPR model outshines the other models in predicting domestic water demand.

### Ecological factors

**Fig 10:**
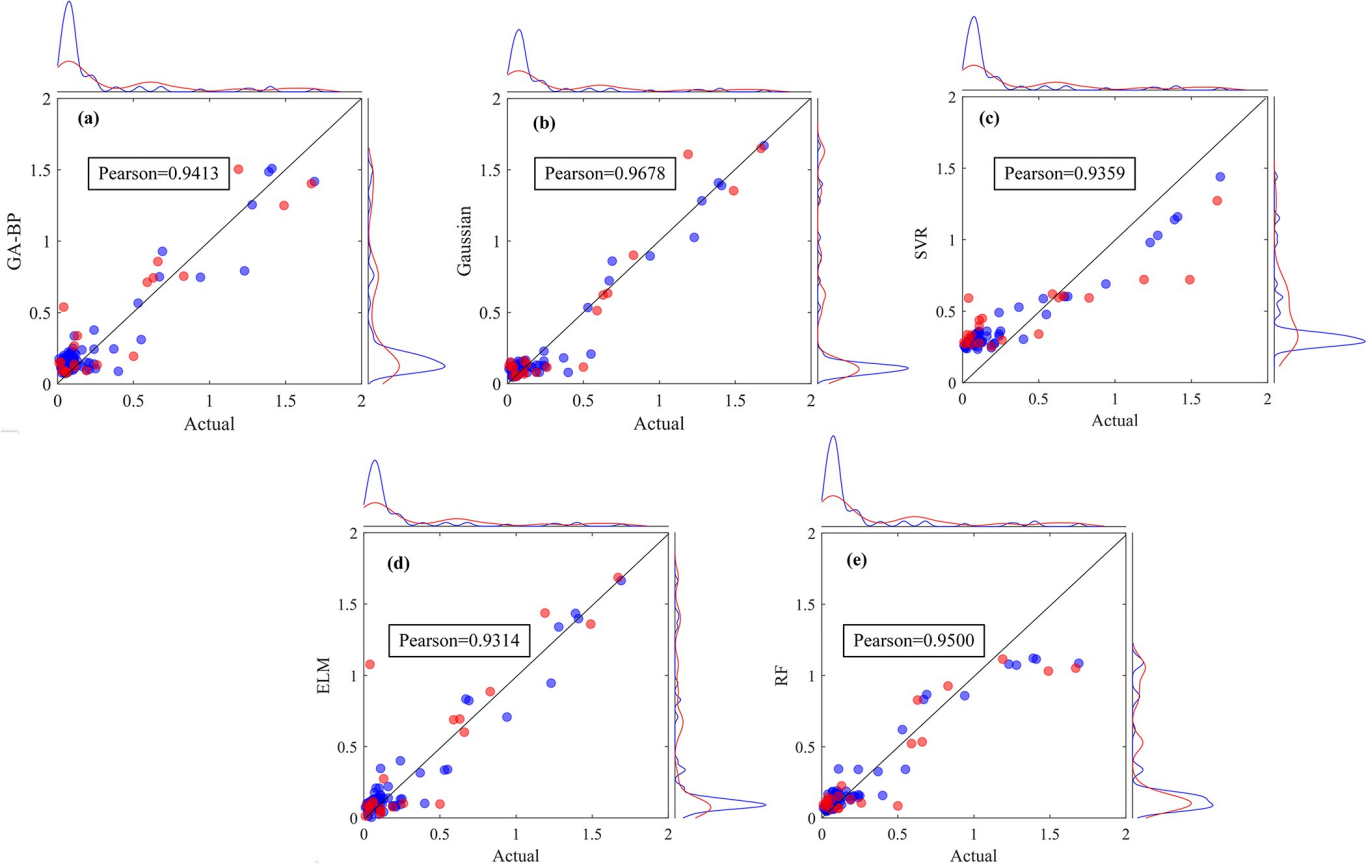
Comparison of predicted and actual values based on (a)GA-BP; (b)GPR; (c)SVR; (d)ELM; (e)RF for ecologial water demand.

Fig 10 illustrates the fitting results from various models used to predict ecological water demand. The optimal model is then chosen for a more in-depth, quantitative evaluation, utilizing certain evaluation indicators (Table 7).

**Table 7:** Performance evaluation results for each model for ecological water demand.

		GA-BP	GPR	SVR	ELM	RF
**Training set**	R^2^	0.8820	**0.9439**	0.6467	0.9267	0.8952
	RMSE	0.1225	**0.0845**	0.2120	0.0965	0.1155
	MAPE	141.1156	79.9659	337.8206	79.2879	**70.8190**
**Test set**	R^2^	0.8464	**0.9142**	0.5856	0.7208	0.8193
	RMSE	0.1928	**0.1442**	0.3168	0.2600	0.2092
	MAPE	206.7609	130.2177	430.9229	185.0726	**118.1022**

Fig 10 shows the same principle as 4.2. The Pearson correlation value of GPR model is the largest, which is 0.9678, followed by RF, GA-BP, SVR and ELM. From the distribution of data points, the SVR model fit is clearly the worst, with a large number of data points having predicted values much lower than the actual values.

From Table 7 it can be seen that the GPR model clearly outperforms the others during the training phase. Even though the GPR model’s MAPE value is not the smallest in the testing stage, both the R^2^ and RMSE values affirm the model’s reliability and accuracy for the test set. This, coupled with the model performance portrayed in Fig 10, confirms that the GPR model is the superior choice for predicting ecological water demand.

### Predicting future water demand

**Fig 11:**
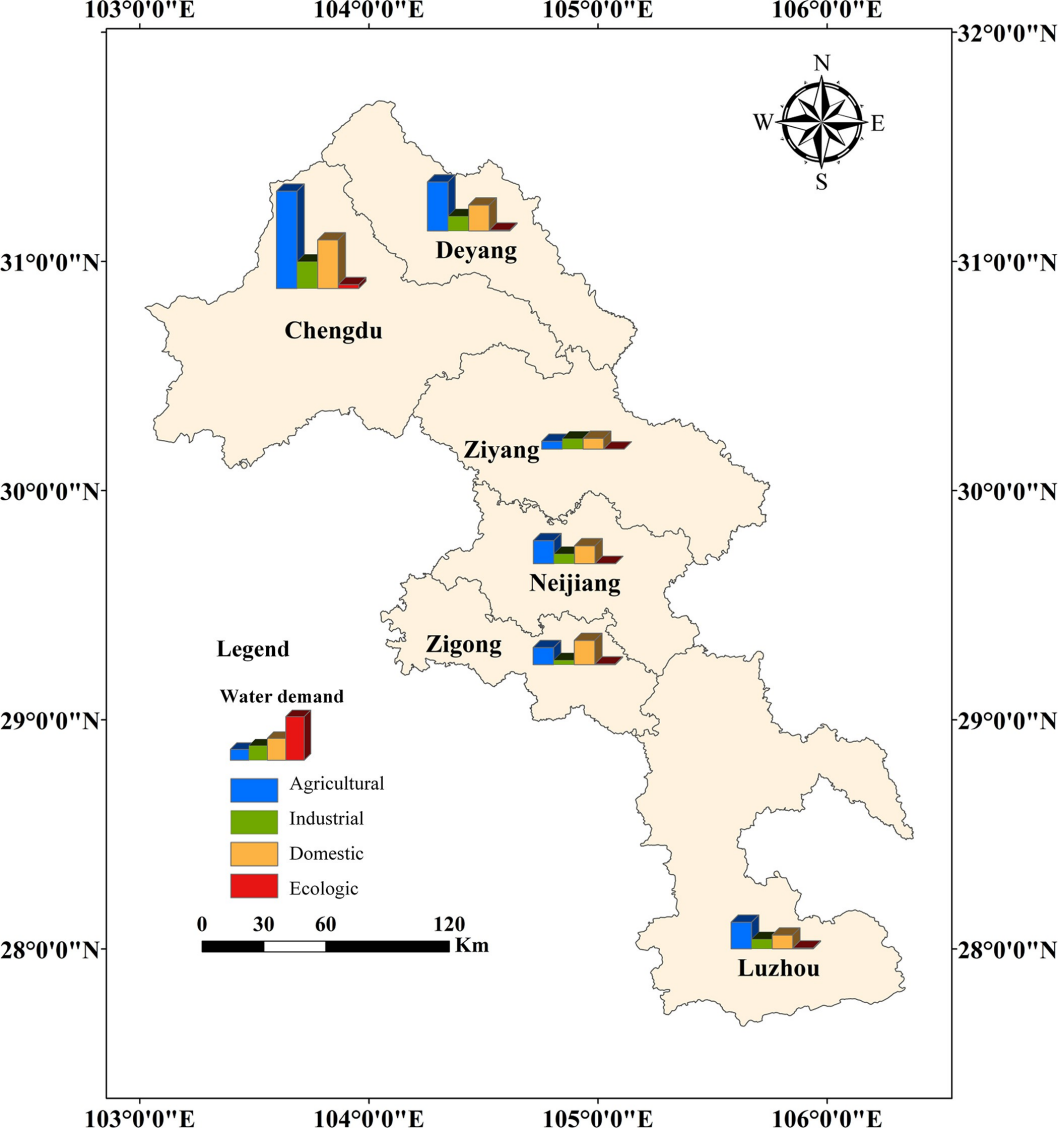
Map of water demand in the Tuo River basin in 2025 ((the map was created by our team using standard maps obtained from the Ministry of Natural Resources of the People’s Republic of China (https://www.mnr.gov.cn/) using ArcMap 10.8)). These maps with approval numbers can be accessed free of charge from the agency’s public website GS(2023)2765 (http://bzdt.ch.mnr.gov.cn /browse.html?picId = %224o28b0625501ad13015501ad2bfc2190%22), Sichuan S(2021)00056 (https://scsm.mnr.gov.cn/StandMaps/mapDetails.html).

Applying the most appropriate water demand forecasting models for each water use sector identified in this study to the 2025 water demand projections for the study area provides decision-makers with reliable, annual water demand estimates per location. This information is crucial for decision-makers to align water resource availability with economic development levels, enabling optimal scheduling of water supply systems. In the SPSS software platform, for the time-series data of each influencing factor in terms of years, three non-seasonal time-series models in the exponential smoothing model were used: Holt linear trend model, Brown linear trend, and decay trend, and based on the model fitting statistic R^2^ and stable R^2^ to determine the most suitable predictive model for each influencing factor to complete the prediction of the input variables of the indicator system in 2025. The previously identified water demand forecasting models for each water use sector were applied individually to determine the agricultural, industrial, domestic, and ecological water demands for the six regions in 2025. These findings are illustrated in Fig 11, with actual water demands for 2020 included for comparative trend analysis (Table 8).

**Table 8:** Comparison of water demand of six regions in 2020 and 2025.

Regions	Agricultural		Industrial		Domestic		Ecological	
	2020	2025	2020	2025	2020	2025	2020	2025
**Chengdu**	28.39	28.31	3.16	8.202	16.59	14.12	1.41	1.14
**Deyang**	12.99	14.21	2.19	4.2688	2.06	7.54	0.55	0.24
**Ziyang**	4.07	2.30	0.21	3.079	1.05	3.17	0.04	0.04
**Neijiang**	4.88	6.73	0.36	2.8597	1.28	5.23	0.02	0.07
**Zigong**	3.83	4.95	0.42	1.2792	1.42	7.04	0.11	0.18
**Luzhou**	6.89	7.74	1.93	2.7865	2.6	3.97	0.11	0.21

Fig 11 reveals that by 2025, Chengdu, as the provincial capital of Sichuan, will have the highest agricultural and domestic water demand in the Tuojiang River basin. The water consumption pattern in Deyang in 2025 will parallel Chengdu, with agricultural and domestic water demand being more significant. Ziyang will demonstrate a more balanced water demand across the three primary water use sectors, albeit at a lower level. Neijiang and Zigong exhibit similar socio-economic levels, with their respective GDP rankings as 10th and 11th within the province in 2022. This similarity is reflected in their comparable water demand and water consumption structures across all sectors. However, due to Neijiang’s industrial development prominence and large-scale industrial expansion, its industrial water demand will be more pronounced. Luzhou’s domestic water demand will be lower than other regions, but its agricultural water demand will be higher, and industrial water demand will align with Ziyang and Neijiang. In terms of ecology, all regions will exhibit relatively low ecological water demand, and specific value changes can be seen in Table 8.

Table 8 reveals that agriculturally, apart from Chengdu and Ziyang, all other regions will witness varying degrees of agricultural water demand growth in 2025. This highlights that the Tuojiang River basin, being a developed agricultural region in Sichuan Province, will continue prioritizing specialty agriculture. Industrial water demand will notably increase across all regions. Consequently, each region must adjust its current development model and improve industrial water-saving efficiency to maximize industrial water conservation and emission reduction. Domestically, Chengdu will experience a decline in domestic water demand as urbanization matures and residents’ water conservation awareness generally increases. In contrast, the other regions will see a significant rise in domestic water demand due to urbanization progression and increased tap water usage in rural areas. Ecologically, only Chengdu and Deyang will experience a minor decrease in ecological water demand, while all other regions will see an increase.

## Discussion

As an indispensable natural resource for human survival and social development, water plays an extremely important role in sustainable socio-economic development [47]. However, increasing water scarcity under the influence of human activities and urbanization is seen as a global systemic risk [48, 49]. In order to develop targeted management measures, water demand forecasting is an important tool to assess the state of water security and to identify possible problems in future water resource systems. Previous forecasting models are mainly suitable for large data, and it is difficult to obtain accurate forecasts for small sample data [8]. This paper compares the applicability of different methodological models to various water use sectors based on short time series data and applies the best model to the 2025 water demand forecast. The results show that agricultural water demand in the Tuo River basin will increase by 2025, indicating an increase in the intensity of agricultural production. In recent two decades, Tuojiang River basin has been the top priority of comprehensive water pollution control, and agricultural and rural non-point source pollution is a major source of pollution in the Tuojiang River basin. Therefore, under the prospect of expanding the scale of agricultural production in the future, cultivating modern and efficient agriculture and improving the ability of agricultural non-point source pollution control are the focus of future exploration of new paths for green development and transformation in the Tuojiang basin, and promoting ecological environmental protection and sustainable economic and social development in the Tuojiang basin. Industrial water use efficiency is an important indicator of industrial water demand and is related not only to the technological heterogeneity of different regions, but also to the control and management of industrial water pollution [50]. In this regard, specific measures such as optimising the industrial layout, promoting new water-saving technologies and limiting the scale of high water-consuming and high-polluting enterprises are needed to ease the burden of water resources on the industrial side. The continued increase in urbanization rates has not only led to a corresponding increase in urban domestic water consumption, but has also led to an increase in impervious area as a result of rapid urbanization to accommodate migration to urban centres [51], resulting in reduced groundwater recharge and increased evaporation of surface water runoff [52]. Consequently, decision makers should take into account other ecological aspects of water security arising from changes in impervious areas while coping with increasing domestic water demand, and minimise the negative ecological impacts of reduced ecological water consumption.

There are two primary areas where this study could be improved: (1) The water demand prediction index applicable to each internal system may vary due to differences in geographical, climatic, economic, and social conditions of each region. If the model proposed in this paper is directly applied to other regions, the problem of low indicator correlation may arise. To augment the universal applicability of the research results, further extraction of general indicators will be pursued. (2) Given that water demand is closely linked to water vulnerability, future research in water forecasting will concentrate on investigating water vulnerability at smaller scales. This will increase the value of regional water demand forecasting in optimizing water operations and planning.

## Conclusions

This paper employed artificial intelligence models to establish long-term, annual water demand forecasting models. Using the six regions along the Tuojiang River basin as the study area, the output data is the actual annual water demand of each water use sector. The input data for agricultural water demand (*AWD*) are Agricultural EVA (*A*_1_), Area of irrigated arable land (*A*_2_), Area of total crop sown (*A*_3_), Percentage of primary industries (*A*_4_). The input data for industrial water demand (*IWD*) are Percentage of secondary industries (*I*_1_), Industrial EVA (*I*_2_), Operating income of industrial enterprises above designated size (*I*_3_), Number of industrial households above designated size (*I*_4_). The input data for domestic water demand (*DWD*) are Per capita GDP (*D*_1_), Population density (*D*_2_), Urbanization rate (*D*_3_), Urban per capita disposable income (*D*_4_), Rural per capita disposable income (*D*_5_). The input data for ecological water demand (*EWD*) are Runoff depth (*E*_1_), Urban green space (*E*_2_), Area of road sweeping and cleaning (*E*_3_), Rainfall (*E*_4_), Average temperature (*E*_5_). The sample size for each water use sector is comprised of the data points from these six regions from 2005 to 2020, amounting to 96 in total. The efficacy of the five forecasting models, which include artificial neural network models (GA-BP, ELM) and regression models (GPR, SVR, RF), is evaluated by comparing the correlation between predicted and actual values in the training and test sets, as well as the statistical evaluation indicator values. The results reveal that the GPR models offer superior performance for agricultural, domestic, and ecological water demand forecasting, while the GA-BP model performs best for industrial water demand forecasting. The optimal model for each water sector is applied to forecast the water demand in 2025 in the study area. The forecast indicates that by 2025, most regions will experience increased water demand across each sector. These forecast results can aid managers in obtaining a clearer understanding of future water use structures and supply and demand trends, allowing them to manage supply and storage in order to optimize distribution costs and maximize socio-economic sustainable development coordination across regions.

## Supporting information

S1 Table It mainly includes data on the following: The water demand and influencing factors data of the Tuojiang River Basin from 2005 to 2020; the predicted values of all models in the training and testing stages; the values of influencing factors and the results of water demand prediction in 2025. (XLSX)
